# MAFin: motif detection in multiple alignment files

**DOI:** 10.1093/bioinformatics/btaf125

**Published:** 2025-03-19

**Authors:** Michail Patsakis, Kimonas Provatas, Fotis A Baltoumas, Nikol Chantzi, Ioannis Mouratidis, Georgios A Pavlopoulos, Ilias Georgakopoulos-Soares

**Affiliations:** Institute for Personalized Medicine, Department of Molecular and Precision Medicine, The Pennsylvania State University College of Medicine, Hershey, PA 17033, United States; Huck Institutes of the Life Sciences, The Pennsylvania State University, University Park, PA 16802, United States; Institute for Personalized Medicine, Department of Molecular and Precision Medicine, The Pennsylvania State University College of Medicine, Hershey, PA 17033, United States; Huck Institutes of the Life Sciences, The Pennsylvania State University, University Park, PA 16802, United States; Division of Basic Sciences, University of Crete Medical School, Heraklion 71110, Greece; Institute for Fundamental Biomedical Research, BSRC “Alexander Fleming”, Vari 16672, Greece; Institute for Personalized Medicine, Department of Molecular and Precision Medicine, The Pennsylvania State University College of Medicine, Hershey, PA 17033, United States; Huck Institutes of the Life Sciences, The Pennsylvania State University, University Park, PA 16802, United States; Institute for Personalized Medicine, Department of Molecular and Precision Medicine, The Pennsylvania State University College of Medicine, Hershey, PA 17033, United States; Huck Institutes of the Life Sciences, The Pennsylvania State University, University Park, PA 16802, United States; Institute for Fundamental Biomedical Research, BSRC “Alexander Fleming”, Vari 16672, Greece; Institute for Personalized Medicine, Department of Molecular and Precision Medicine, The Pennsylvania State University College of Medicine, Hershey, PA 17033, United States; Huck Institutes of the Life Sciences, The Pennsylvania State University, University Park, PA 16802, United States

## Abstract

**Motivation:**

Whole Genome and Proteome Alignments, represented by the multiple alignment file format, have become a standard approach in comparative genomics and proteomics. These often require identifying conserved motifs, which is crucial for understanding functional and evolutionary relationships. However, current approaches lack a direct method for motif detection within MAF files. We present MAFin, a novel tool that enables efficient motif detection and conservation analysis in MAF files to address this gap, streamlining genomic and proteomic research.

**Results:**

We developed MAFin, the first motif detection tool for Multiple Alignment Format files. MAFin enables the multithreaded search of conserved motifs using three approaches: (i) using user-specified k-mers to search the sequences. (ii) with regular expressions, in which case one or more patterns are searched, and (iii) with predefined Position Weight Matrices. Once the motif has been found, MAFin detects the motif instances and calculates the conservation across the aligned sequences. MAFin also calculates a conservation percentage, which provides information about the conservation levels of each motif across the aligned sequences, based on the number of matches relative to the length of the motif. A set of statistics enables the interpretation of each motif's conservation level, and the detected motifs are exported in JSON and CSV files for downstream analyses.

**Availability and implementation:**

MAFin is offered as a Python package under the GPL license as a multi-platform application and is available at: https://github.com/Georgakopoulos-Soares-lab/MAFin.

## 1 Introduction

The increase in the number of available organismal genomes and of individual human genomes, in part facilitated by multiple international consortia (Exposito-Alonso *et al.* 2020, Karczewski *et al.* 2020, Rhie *et al.* 2021, Darwin Tree of Life Project Consortium 2022), requires advanced and scalable algorithms to derive the most useful information from the generated sequences.

Sequence relatedness between genomes is an active research area that potentiates the identification of conserved elements, regions undergoing accelerated evolution, and divergent sequences (Bejerano *et al.* 2004, Pollard *et al.* 2006, Alkan *et al.* 2011). Multiple Sequence Alignment (MSA) enables the alignment of two or more related sequences, to quantify sequence similarity. The alignment output includes mutations that differentiate the sequences, such as substitutions, insertions, and deletions incorporated with alignment gaps. Several different sequence alignment algorithms have been developed (Earl *et al.* 2014, Armstrong *et al.* 2020) and are implemented to generate phylogenetic trees (Liu *et al.* 2010, Zhang *et al.* 2018), perform comparative annotation (Fiddes *et al.* 2018), infer the evolution of species (Misof *et al.* 2014), the evolution of proteins (Caetano-Anollés and Caetano-Anollés 2003), estimate the age of a gene (Dunn *et al.* 2013), find high confidence transcription factor binding sites (Daily *et al.* 2011), and identify clinically relevant mutations (Bao *et al.* 2008), among various applications.

Motifs refer to short patterns in DNA, RNA, or protein sequences that carry biological information (Moeckel *et al.* 2024). These motifs often reflect functionally important sites involved in regulating gene expression, such as transcription factor binding sites (Georgakopoulos-Soares *et al.* 2023), and sites associated with protein function and localization (Vazquez *et al.* 1993). Several motif detection tools have been developed such as The Meme Suite (Grant *et al.* 2011, Bailey *et al.* 2015) and HOMER (Heinz *et al.* 2010), aiming to find motifs in individual sequences. Additionally, motif databases such as JASPAR (Rauluseviciute *et al.* 2024), enable the aggregation of biological motifs, often stored in k-mer or Position Weight Matrix (PWM) formats, while in certain cases motifs are also stored as regular expressions (Todd *et al.* 2005). Additionally, sequence conservation is usually precalculated for alignment files of species based on their taxonomic subdivision, with tools such as phyloP (Pollard *et al.* 2010) and phastCons (Siepel *et al.* 2005). However, even though MSA files are becoming increasingly more utilized across different research problems and applications, no bioinformatics tool enables the detection of motif instances and their conservation directly from MSA files.

Here, we developed MAFin, the first motif detection tool for multiple alignment files (MAFs). MAFin identifies motifs across multiple sequences by comparing the aligned sequences in MAF format. MAFin takes as input a file of k-mer motif sequences, PWMs, or regular expression patterns and an MSA file in the Multiple Alignment Format. Its output is in JSON and CSV formats containing motif coordinates, similarity vectors, conservation percentages, and multiple related diagrams.

## 2 Methods

MAFin is offered as a command line program, accommodates the detection of motifs in MAF files, identifies and stores all motif instances in the alignment file, and produces multiple summary statistics and visualization options. The motif discovery can be performed for the reference sequence in the MSA file or across all sequences. The reference sequence refers to the primary genome or protein sequence used as a baseline for comparison in the multiple sequence alignment stored in the MAF file. It is the sequence against which other aligned sequences are compared to identify variations, motif conservation, and alignment statistics.

MAFin calculates the conservation of the identified motifs across the aligned sequences. The process is based on comparing the gapped sequences within the alignment while preserving the alignment structure in the MAF file. The comparison results in a similarity vector that matches the true length of the ungapped motif, excluding positions where both the reference and compared sequences have gaps. The conservation percentage for each motif instance is calculated by counting the percentage of matched base pairs. The total conservation percentage for a motif within a block is then estimated by calculating the average conservation percentages of all motif instances in the genomes present in the block.

The similarity vector is calculated as follows. For each aligned pair of characters between the reference and the compared genome in the identified motif:

1. If both are gaps, ignore that position.
2. If the reference is a gap and is compared to a character, we record a “–” in the similarity vector.
3. If the reference is not a gap and matches the compared character, record a “1”, for matching.
4. If the reference is not a gap and does not match the compared character (including when the compared character is a gap), record a “0,” for a mismatch.

The conservation core is calculated as the ratio of the positions that have “1” over the total positions including gaps and “0.”

### 2.1 Algorithmic process

**Figure 1.**
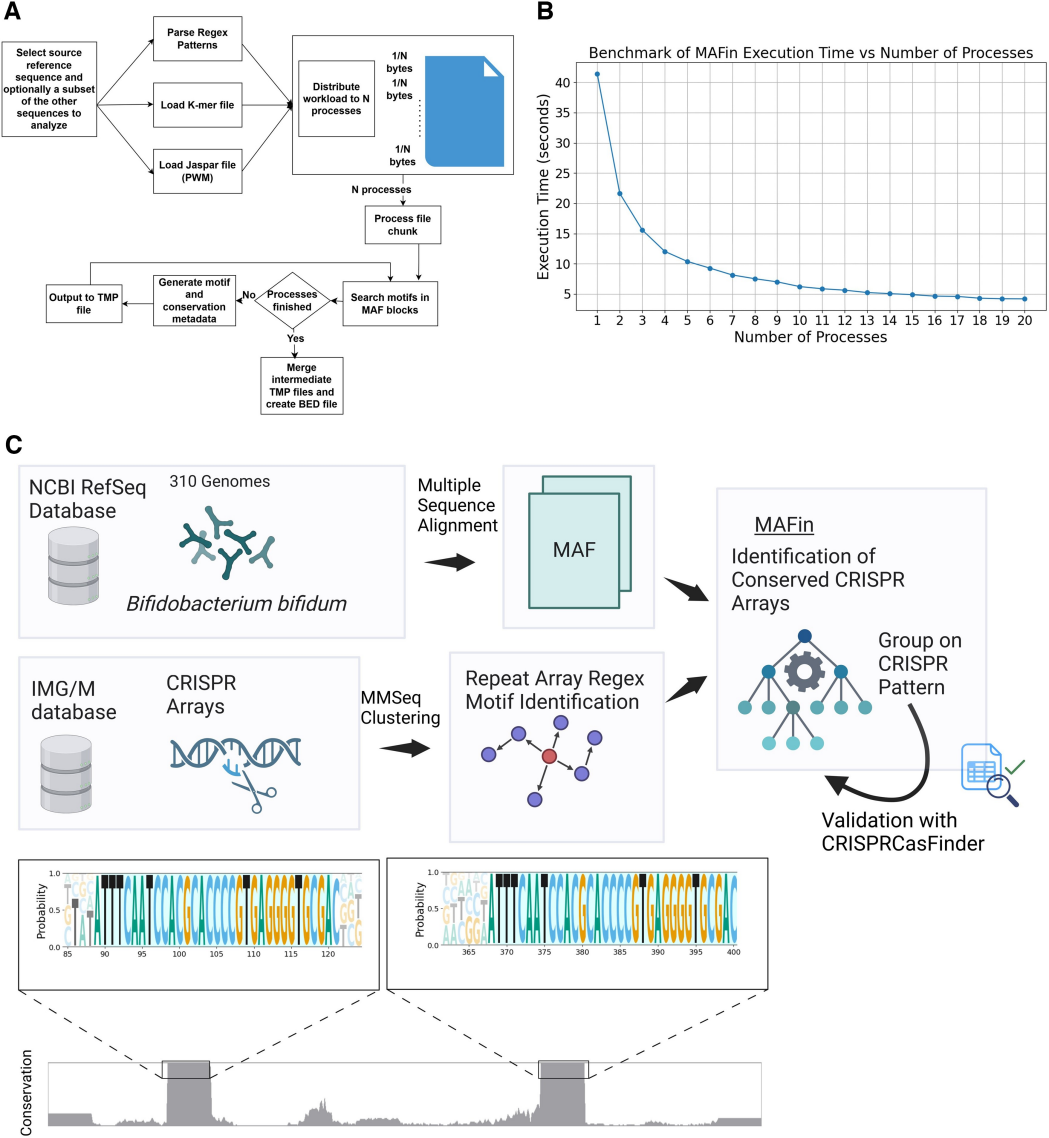
Workflow and performance of MAFin. (A) Workflow Diagram: this diagram illustrates the processes and deployment of MAFin. (B) Execution time: this section shows the execution time given the number of processes utilized. (C) CRISPR array detection: this part demonstrates the detection of CRISPR arrays in genome alignments using MAFin, including an illustration of the developed pipeline and an example of a CRISPR array along with its conservation across *B. bifidum* strains.

MAFin handles the MAF file by dividing it into chunks that are distributed across several processes to take advantage of parallel computation. Each process is responsible for a particular section of the file, where it parses MAF blocks and looks for motifs within the designated genomes (Fig. 1A). At present, MAFin does not allow motif searches across multiple blocks; all searches are conducted independently within each block.

#### 2.1.1 k-mer searching with Aho-Corasick algorithm

MAFin leverages the Aho-Corasick algorithm for k-mer searches (Aho and Corasick 1975). This string-searching technique efficiently processes multiple patterns concurrently in linear time relative to the length of the text. This methodology substantially enhances the speed of pattern matching in large sequences by constructing a finite automaton that encapsulates all k-mers, facilitating the rapid detection of motif occurrences without requiring individual searches for each k-mer.

#### 2.1.2 PWM searching

In PWM searches, the tool determines threshold scores aligned with a specified *P*-value by sampling random sequences that reflect the background nucleotide frequencies. It then scans the sequences with PWMs, reporting positions that surpass the threshold as motif hits.

#### 2.1.3 Regular expression searching

For regular expression searches, conventional regular expression matching techniques are used to detect motifs within the sequences.

MAFin requires the following inputs: an MSA file as a required positional argument, and a motif file, which can be in the form of a file of k-mer motif sequences or a file with PWM motifs in JASPAR format or a set of regular expressions, which are comma-separated.

Optional inputs for the tool include parameters for specifying source IDs within the MAF file to scan for motif matches. By default, the search is conducted in the reference genome sequence, but users can set it to a specific source ID for targeted searching. A file listing genome IDs can also be provided; if omitted, all source IDs will be considered for motif conservation analysis. Users have the option to include the reverse complement in the search. The number of processes used can be adjusted as needed. For the JASPAR motif discovery feature, the threshold for motif matching is automatically set to a *P*-value of 1e-4. Lastly, users can specify background nucleotide frequencies for A, C, G, and T, accepted as four floating-point numbers summing to 1; if not provided, uniform background frequencies are assumed.

The outputs consist of a default BED formatted file and two optional JSON and CSV files that detail the coordinates of discovered motifs, their similarity vectors, and conservation percentages. The JSON file structures its data using key-value pairs, where each key represents an identified motif. These keys contain information such as the source sequence name and motif coordinates, with motifs detected in gapped and ungapped formats. The values provide conservation data for each identified motif, expressed as a binary similarity vector with a length equal to the matched motif, where zero indicates a mismatch and one indicates conservation. Specifically, the JSON file records details such as the source name, chromosome, start and end coordinates, strand, type of motif used (whether PWM, regular expression, or *k*-mer), motif length, motif sequence, gapped and ungapped start and end positions from the MAF file block, conservation statistics, and, for PWM searches, the score, *P*-value, and false discovery rate.

The MAF format is among the most widely used MSA formats (Raney *et al.* 2024) and is the required format for MAFin. MAF files can be generated from other formats, such as the Hierarchical Alignment (HAL) format, using the hal2maf conversion tool (Hickey *et al.* 2013).

## 3 Results

### 3.1 Performance testing

A benchmark was conducted to analyze the performance of MAFin when using multiple processes. Figure 1B shows the execution time as the number of processes increases linearly (from 1 to 20). The results indicate that execution time is inversely proportional to the number of processes.

### 3.2 Case study: rapid detection of CRISPR arrays in genome alignments across hundreds of genomes

CRISPR-Cas is an immunity mechanism of bacteria and archaea to battle infection by viruses, phages, foreign plasmids, and other potentially harmful mobile genetic elements (Shmakov *et al.* 2017). In prokaryotes with active CRISPR systems, infection by a new phage triggers the CRISPR defense mechanism. Cas endonucleases cleave the viral genome into fragments, which are stored as spacers in CRISPR arrays, and upon re-infection, the CRISPR-Cas complexes target and cleave matching viral sequences. Till now, several CRISPR system types and subtypes have been reported (Koonin and Makarova 2019) and various CRISPR detection algorithms have been implemented (Clement *et al.* 2020). However, their large-scale application faces challenges related to scaling and memory demands, making motif detection advantageous due to the conserved nature of CRISPR array motifs.

To showcase the practical capabilities of MAFin, we have used its motif search method to detect the positions of CRISPR arrays in different strains of *Bifidobacterium bifidum*, a probiotic bacterium commonly found in the mammalian gut microbiome. Like many members of the Bifidobacteria family, multiple *B. bifidum* strains have been found to have beneficial effects on the host, including protection against pathogens, regulation of immune responses, and balancing the gut microbiota of newborns (Turroni *et al.* 2019). Previous studies indicate that the interactions between *B. bifidum* and its infecting phages regulate many effects and may drive population adaptation and host viability (Ventura *et al.* 2009, 2010, Briner *et al.* 2015). Thus, examining the diversity of CRISPR systems in *B. bifidum* strains is crucial for understanding these phage interactions.

The first step was to construct a multiple genome alignment of *B. bifidum* different strain genomes. Toward this end, we retrieved a total of 310 different *B. bifidum* genome assemblies from NCBI RefSeq (Goldfarb *et al.* 2025). Whole genome alignment was performed using SibeliaZ (Minkin and Medvedev 2020), and the results were saved in the MAF format. The next step was to conduct searches for patterns matching the structure of CRISPR arrays ([repeat—spacer—repeat]*N, where N can be ≥ 1). To identify repeat sequences, we performed searches in the Integrated Microbial Genomes & Microbiomes (IMG/M) database (Chen *et al.* 2023) for microbiome datasets of *B. bifidum* containing information on CRISPR arrays, extracted their corresponding repeat sequences, and clustered them based on their sequence identity using MMseqs2 (Steinegger and Söding 2017), with a threshold of 95% (allowing for one or two substitutions per sequence). These resulted in a total of 11 different potential repeat sequence clusters, each represented by a consensus motif. The motifs were adjusted to also include the existence of spacers corresponding to viral genomic fragments (protospacers), the spacer length ranging from 28 to 36 bps, in line with previous work on *B. bifidum* CRISPR elements (Briner *et al.* 2015). Each of the different motifs was searched as a regular expression pattern against the MAF alignment using MAFin, on a Linux workstation with 8 CPU cores and 32 GBs of RAM. The results were filtered to discard any false positive hits containing only a single copy of the repeat sequence without spacers, and the filtered results were grouped based on their corresponding CRISPR pattern and aligned using MAFFT (Katoh *et al.* 2002).

A total of 30 different genomes were found containing valid CRISPR arrays, corresponding to 3 different motifs. These results were verified by running the sequences of the respective genomes using CRISPRCasFinder (Couvin *et al.* 2018); all of the CRISPR arrays were reported by the latter method in the same positions identified by MAFin, thus validating the procedure used. The identified arrays contain multiple spacers surrounded by repeats; multiple sequence alignments (MSAs) of the results show that the MSA regions corresponding to the arrays are highly conserved, while the interjected spacer sequences can be very divergent (Fig. 1C); this would indicate that the contained spacers likely target different phages.

Notably, the average execution time of MAFin searching each regex pattern against all genomes in the MAF alignment ranged from 4.2 to 5.8 s; this indicates that even though the size of the alignment is large (310 genomes), the search, as performed by the method, is very fast. In comparison, CRISPRCasFinder takes on average, 20 s to analyze a single genome. We conclude that MAFin can be used to rapidly and effectively detect CRISPR arrays in bacterial genomes.

### 3.3 Dependencies and libraries

The tool is developed in Python and uses several external libraries to enhance its functionalities. It specifically relies on Biopython for efficient parsing of sequence alignments, NumPy for numerical computations, and pyahocorasick for fast k-mer searching (Cock *et al.* 2009, Harris *et al.* 2020, Muła). All these libraries are open-source and can be conveniently installed using standard package managers.

## 4 Discussion

This work introduces MAFin, the first motif discovery tool specifically designed for multiple alignments. With the increasing number of sequenced genomes, the use of alignments for comparative analysis is becoming more prevalent, and this trend is likely to continue. MAFin offers a user-friendly and efficient means of discovering motifs within MAF files and assessing the conservation of each motif instance. It generates JSON and CSV outputs for each source sequence name or species in the alignment file, along with summary statistics and visualizations. Given the growing number of applications utilizing MSA files such as comparative annotation, species identification, phylogenetic tree construction, and functional studies, MAFin facilitates the detection of sequences of interest and functional elements and their conservation levels within these alignments.

## Supplementary Material

btaf125_Supplementary_Data

## Data Availability

The GitHub code and all the related material is provided at: https://github.com/Georgakopoulos-Soares-lab/MAFin. A stable version is also found at: https://zenodo.org/records/14977168.
