# Supplementary material for: MAFin: motif detection in multiple alignment files
Source: Bioinformatics. 2025 Mar 19;41(4):btaf125. doi: 10.1093/bioinformatics/btaf125 (PMC11978385; doi:10.1093/bioinformatics/btaf125)
Supplement: btaf125_Supplementary_Data [file btaf125_supplementary_data.zip › Supplementary Material MAFin.pdf]

Supplementary Material

Examples

Example of Conservation logic

The example covers all edge cases when applying the conservation logic algorithm between a pair of sequences.

Motif: ATCGAC

Reference Genome: A – T C G A – C

Compared Genome: A G T G – A – C

The process is completed following the algorithm described in the Materials and methods section.

Step-by-Step Comparison:

**Position**	**1**	**2**	**3**	**4**	**5**	**6**	**7**	**8**
**Ref Base**	A	–	T	C	G	A	–	C
**Other Base**	A	G	T	G	–	A	–	C
**Result**	Match	Gap	Match	Mismatch	Mismatch	Match	Skip	Match
**Vector**	1	–	1	0	0	1		1

Similarity Vector:

Positions 1, 3, 6, and 8 yield a “1” (match). Positions 4 and 5 yield a “0” (mismatches), and position 2 is recorded as “–” (gap–nucleotide). The gap–gap at position 7 is skipped. Thus, the resulting similarity vector is: [1,-,1,0,0,1,1]

Conservation Percentage:

Here, the vector length is 7 (position 7 is skipped), and the number of matches (i.e. “1”s) is 4. Therefore, the conservation score is computed as:

$$\text{Conservation}=4/7\times100\%\approx57.14\%$$

Genomic Coordinates for Example:

In addition to the similarity vector, MAFin supplies the genomic coordinates of the motif. Consider the example above (using 0-indexed coordinates): After comparing the sequences (where gaps in both sequences are skipped), the ungapped reference motif is: A, T, C, G, A, C (a total of 6 bases). If this motif begins at position 1000 (0-indexed) in the reference genome, then its genomic coordinates are reported as:

Start: 1000, End: 1005

Example of Reverse Complement Searches

Searching for a motif on the reverse strand typically requires looking for the reverse complement of the sequence.

Searching reverse strand through Regex Patterns

Reference Sequence: ATCGGCA

Regular Expression: C{2}G (Two times C followed by G)

Given the complex nature of Regex patterns, it is not feasible to reverse and complement the expression itself. Therefore, MAFin reverses and complements the reference sequence and subsequently searches for the pattern within that modified sequence.

Reverse complement match of CCG in sequence: TGCCGAT results in genomic coordinates 3,5:

**1**	**2**	**3**	**4**	**5**	**6**	**7**
T	*G*	*C*	*C*	*G*	*A*	*T*

Searching reverse strand through K-mers

Reference Sequence: ATCGGCA

K-mer: CCG

Searching for a *k*-mer on the reverse strand is considerably simpler. In such a case, MAFin simply reverses and complements the *k*-mer sequence and then searches for it on the original strand.

Reverse complement K-mer: CGG is found at genomic coordinates 3,5:

**1**	**2**	**3**	**4**	**5**	**6**	**7**
A	*T*	*C*	*G*	*G*	*C*	*A*

Searching reverse strand through PWMs (JASPAR format)

PWM:

**Position**	1	2	3	4
A	0.2	0.1	0.4	0.3
C	0.3	0.5	0.1	0.2
G	0.4	0.3	0.4	0.5
T	0.1	0.1	0.1	0.0

To search for a motif on the reverse strand, it is necessary to compute the reverse complement of the PWM. This involves reversing the order of the positions and replacing each nucleotide with its complement. Consequently, the reverse PWM is represented as follows:

Reverse PWM:

**Position**	1	2	3	4
A	0.0	0.1	0.1	0.1
C	0.5	0.4	0.3	0.4
G	0.2	0.1	0.5	0.3
T	0.3	0.4	0.1	0.2

The standard process of PWM search is shown in the workflow diagram (**Figure 1a**).
